# Simultaneous Inference of Multiple Binary Endpoints in Biomedical Research: Small Sample Properties of Multiple Marginal Models and a Resampling Approach

**DOI:** 10.1002/bimj.202300197

**Published:** 2024-07-02

**Authors:** Sören Budig, Klaus Jung, Mario Hasler, Frank Schaarschmidt

**Affiliations:** ^1^ Department of Biostatistics Institute of Cell Biology and Biophysics Leibniz University Hannover Hannover Germany; ^2^ Institute for Animal Breeding and Genetics University of Veterinary Medicine Hannover Hannover Germany; ^3^ Lehrfach Variationsstatistik Christian‐Albrechts‐University of Kiel Kiel Germany

**Keywords:** bootstrap, family‐wise error rate, generalized linear models, multiple comparisons, power

## Abstract

In biomedical research, the simultaneous inference of multiple binary endpoints may be of interest. In such cases, an appropriate multiplicity adjustment is required that controls the family‐wise error rate, which represents the probability of making incorrect test decisions. In this paper, we investigate two approaches that perform single‐step p‐value adjustments that also take into account the possible correlation between endpoints. A rather novel and flexible approach known as multiple marginal models is considered, which is based on stacking of the parameter estimates of the marginal models and deriving their joint asymptotic distribution. We also investigate a nonparametric vector‐based resampling approach, and we compare both approaches with the Bonferroni method by examining the family‐wise error rate and power for different parameter settings, including low proportions and small sample sizes. The results show that the resampling‐based approach consistently outperforms the other methods in terms of power, while still controlling the family‐wise error rate. The multiple marginal models approach, on the other hand, shows a more conservative behavior. However, it offers more versatility in application, allowing for more complex models or straightforward computation of simultaneous confidence intervals. The practical application of the methods is demonstrated using a toxicological dataset from the National Toxicology Program.

## 1 Introduction

In many studies, the outcome variables are several discrete binary variables and it is often of interest to evaluate several hypotheses simultaneously. For example, in toxicology, carcinogenicity studies are conducted to investigate the effect of increasing doses of a chemical on the presence or absence of different tumor types (Ishii et al. 2017; NTP 2019; Yamaguchi et al. 2023). When new drugs are investigated in pharmacological or clinical trials, the presence or absence of several types of adverse events at the patient level is recorded (Long et al. 2018; Whone et al. 2019). Testing hypotheses for each of these endpoints separately, without adjusting the p‐values, will inflate the overall type I error. This results in an increased probability of incorrectly reporting an effect of the drug or chemical for at least one of the binary endpoints.

Many methods for analyzing multiple binary endpoints have been investigated, but the focus has mainly been on comparing two groups (Agresti and Klingenberg 2005; Klingenberg and Satopää 2013; Leon and Heo 2005; Ristl et al. 2018), using combined tests (Whitehead, Branson, and Todd 2010) or composite endpoints (Rauch and Kieser 2012). In addition, there are a number of closed testing procedures, including step‐down (Holm 1979; Holland and Copenhaver 1988) and step‐up (Hochberg 1988; Hommel 1988; Simes 1986) procedures. A comprehensive summary of the methods available in 2016 for the simultaneous inference of multiple endpoints, including binary endpoints, can be found in Ristl et al. (2018). Validation studies of methods that allow the analysis of models for multiple binary data, which can also include more complex experimental structures, and at the same time are relatively easy to use, were not found.

Therefore, the aim of this paper was to compare and evaluate two model‐based approaches that allow the simultaneous analysis of multiple, possibly correlated, binary endpoints. First, we investigated the approach of Pipper, Ritz, and Bisgaard (2012), which allows a single‐step adjustment of p‐values by approximating the joint distribution of the parameter estimates for marginal models of interest. The advantages of this approach are that it is implemented in the R environment (R Core Team 2021) in the multcomp package (Hothorn, Bretz, and Westfall 2008), it is easy to use, and it allows the simultaneous evaluation of different scaled endpoints or models of varying complexity. For example, it is straightforward to include covariates in the models for some or all binary endpoints or to account for differences between strata or subgroups such as sex or age groups. In addition to adjusted p‐values, simultaneous confidence intervals can be computed for the effect parameter of each endpoint. This allows the toxicological or clinical relevance of observed effects to be assessed in addition to statistical significance.

As an alternative method for single‐step p‐value adjustment, we also investigated the minP bootstrap approach of Westfall and Young (1989), which has been published for some time, but its performance with small sample sizes has not yet been investigated. There is also uncertainty about how and to what extent the method can be applied to more complex models. In a simulation study, using single slope regression models and group comparison models, we investigated the performance of these two approaches in terms of family‐wise error rate (FWER) and power and compared them with each other and also with the well‐known but simplistic Bonferroni adjustment. In general, the focus of this paper is on datasets with a low‐to‐moderate number of endpoints, and the application of adjustments to high‐dimensional datasets has not been investigated.

When studying rare events, a high proportion of zeros in the binary data can lead to extreme estimates of the standard errors. This can lead to technical problems in the multiple marginal models (MMM) approach. Bayesian generalized linear models with weakly informative priors lead to more stable estimates in these situations (Gelman et al. 2008). Therefore, we additionally investigated their performance using the three methods.

In the remainder of this paper, we first describe the MMM approach for models with multiple binary outcomes and recall the minP adjustment of Westfall and Young (1989) for this application. Second, simulation studies for typical sample sizes of toxicological studies compare these methods in terms of FWER and power. Finally, the application is illustrated using a data example from a long‐term rodent carcinogenicity study.

## 2 Methods

### 2.1 Multiple Endpoints and Marginal Generalized Linear Models

Consider a study that examines the occurrence of binary events at several different endpoints j=1,…,J in individuals i=1,…,N. If it is of interest to identify a dose–response relationship with at least one of the binary events, different dose levels of a substance of interest may be randomly assigned to the N individuals. Often, only a few dose levels are considered, each assigned to several individuals. In the following, we consider two different options for the systematic part of the models: First, regression analyses can be performed to assess the effect of increasing dose on the frequency of events at endpoint j. In this case, one slope parameter is estimated for each endpoint j. Second, with replicates for each dose group, it is also possible to estimate a dose‐specific parameter to compare higher dose groups with a control group, analogous to Dunnett's test. Multiple parameters are then estimated for each of the j endpoints. The following models allow for both options and are set up as marginal models, that is, they are fitted separately for each of the j endpoints. We obtain an (N×J) matrix Y of binary data, with the elements $y_{ji}$ recording the presence (1) or absence (0) of an event at endpoint j in individual i. The elements $y_{ji}$ are assumed to follow binomial distributions $y_{ji}∼B\left(1,\pi_{ji}\right)$, where $\pi_{ji}$ denotes the corresponding event probabilities. To test the effect of dose levels for a particular endpoint j, we assume that the following generalized linear model (GLM) may be appropriate:

$$g\left(\pi_{j}\right)=X_{j}\beta_{j},$$

where g is the link function, $\pi_{j}={\left(\pi_{j1},\text{…},\pi_{jN}\right)}^{T}$ is a column vector with index i=1,…,N, $X_{j}$ is the design matrix consisting of N rows and the number of explanatory variables as columns and $\beta_{j}$ is a column vector containing the parameters of interest. Although the single‐step p‐value adjustment methods described below are more generally applicable, the canonical logit link $g\left(\pi_{ji}\right)=log\left(\pi_{ji}/\left(1-\pi_{ji}\right)\right)$ is used in the following. In the given situation, a univariate GLM can now be fitted for each individual endpoint, using either a regression model to test the slope parameter of the dose or a group comparison model that allows to compare the dose groups with the untreated control. Derived from the matrix notation, we first consider the simple regression model for the J endpoints:

$$g\left(\pi_{ji}\right)=\beta_{j0}+\beta_{j1}x_{i},$$

where $x_{i}$ is the covariate vector containing the dose administered to individuals i=1,…,N. Here the parameter of interest is $\beta_{j1}$ and the corresponding hypotheses of interest can be formulated as

$$H_{0jk}:\beta_{jk}=0,\;\text{for all}\;j=1,\text{…},J,\;k=1,$$

$$H_{Ajk}:\beta_{jk}\neq 0,\;\text{for at least one}\;j=1,\text{…},J\;k=1.$$

The index k=0,…,K indicates which parameter is being considered, and since we only have one parameter here that reflects the effect of dose we set k=1 to test the slope in the regression model.

Alternatively, each dose group may be compared to the untreated control group, in analogy to a Dunnett's test. This can be achieved by dummy‐coding the dose levels in $X_{j}$:

$$g\left(\pi_{ji}\right)=\beta_{j0}+\beta_{j1}x_{1i}+\beta_{j2}x_{2i}+\cdots +\beta_{jK}x_{Ki},$$

where $\beta_{j0}$ is the parameter for the control group k=0 and the remaining $\beta_{jk}$ with k=1,…,K represents the difference between the dose levels and the control group k=0 and $x_{1},\text{…},x_{K}$ are the respective dummy variables for the dose groups. The following hypotheses can be stated regarding the comparison with control:

$$H_{0jk}:\beta_{jk}=0,\;\text{for all}\;j=1,\text{…},J,\;k=1,\text{…},K,$$

$$H_{Ajk}:\beta_{jk}\neq 0,\;\text{for at least one}\;j=1,\text{…},J,\;k=1,\text{…},K.$$

Unadjusted tests can be performed separately for each endpoint j=1,…,J using the test statistic $t_{jk}={\hat{\beta }}_{jk}/\hat{\mathrm{se}}\left({\hat{\beta }}_{jk}\right)$, where the model parameters ${\hat{\beta }}_{jk}$ are maximum likelihood estimates and $\hat{\mathrm{se}}\left({\hat{\beta }}_{jk}\right)$ is the corresponding model‐based standard error. Within the regression model, the analysis involves testing the slope with k=1 and within the group comparison model the mean differences between the dose groups and the control group are assessed with k=1,…,K. The corresponding asymptotic unadjusted p‐values for endpoint j are

$$p_{jk}=P(|Z|>|t_{jk}\left|\right),$$

where Z is a standard normal random variable with Z∼N(0,1). Since multiple hypotheses are tested simultaneously, inflation of the type I error is expected. Therefore, a multiplicity adjustment is necessary. One approach is to control for the FWER, which is the probability of falsely rejecting at least one $H_{0jk}$ when examining a whole family of JK tests. Two types of FWERs are considered here. First, the FWER can be controlled in a weak sense (FWERW). This means that the FWER is controlled when all hypotheses are under the null, but not under other configurations. Second, the FWER can be controlled in a strong sense (FWERS). In this case, the type I error does not exceed a prespecified α, regardless of how many hypotheses are under the null or the alternative.

An adjustment that controls for the FWER in the strong sense, and which is still widely used today due to its simplicity, is the Bonferroni method (Nomura et al. 2020; Wason, Stecher, and Mander 2014). Here, the hypothesis $H_{0jk}$ is rejected if the p‐value ${\tilde{p}}_{jk}^{Bonf}=\min\left(JKp_{jk},1\right)$ is less than the prespecified α. The disadvantage is that a possible correlation of the individual endpoints is not taken into account, and the procedure is therefore considered to be conservative.

In the following, we present the two methods investigated in this paper, which are expected to provide a p‐value adjustment that controls for the FWER in both the weak and strong sense, and which may offer an advantage over the Bonferroni method in terms of power. All three methods are single‐step adjustments, so the order in which the hypotheses are tested is not important.

### 2.2 Multiple Marginal Models

One way to simultaneously evaluate multiple binary endpoints would be to use MMM. This approach was introduced by Pipper, Ritz, and Bisgaard (2012), and below we will recall the basic results of their work in the context of our application. The approach is based on approximating the joint distribution of the stacked parameter vector $\hat{\beta }=\left({\hat{\beta }}_{11},\text{…},{\hat{\beta }}_{JK}\right)$ by a multivariate normal distribution, where ${\hat{\beta }}_{jk}$ is the maximum likelihood estimator of the unknown parameter $\beta_{jk}$. They showed that ${\hat{\beta }}_{jk}$ satisfies the asymptotic property, which can be carried over to the stacked asymptotic representation

$$\left(\hat{\beta }-\beta \right)\sqrt{N}=\frac{1}{\sqrt{N}}\sum_{i=1}^{N}\Psi_{i}+op\left(1\right),$$

where $\beta =(\beta_{11},\text{…},\beta_{JK})$, $\Psi_{i}=\left(\Psi_{1i},\text{…},\Psi_{Ji}\right)$, and $\Psi_{ji}=-I_{j}^{-1}{\tilde{\Psi }}_{ji}$, where $I_{j}^{-1}$ is the row in the inverse Fisher information matrix of $\beta_{jk}$, ${\tilde{\Psi }}_{ji}$ is the score function for the ith individual, and op(1) indicates a sequence of random vectors that converges to zero in probability. Note that stacking does not destroy independence. The multivariate central limit theorem ensures the convergence of the left‐hand side of the equation


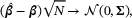

where by the weak law of large numbers the empirical variance–covariance matrix of the stacked standardized score contributions converges in probability to the matrix Σ:


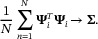

Thus, the covariance Σ can be estimated by

$$\hat{\Sigma }=\frac{1}{N}\sum_{n=1}^{N}{\hat{\Psi }}_{i}^{T}{\hat{\Psi }}_{i},$$

where the ${\hat{\Psi }}_{i}$ are obtained by using the parameter estimates from the J models of the respective endpoints. By standardizing $\hat{\Sigma }$ by its diagonal elements, we obtain an asymptotic estimate of the correlation matrix $\hat{R}$ for the JK test statistics of interest.

Note that the package multcomp, which is used for the subsequent simulation study, uses an alternative calculation for $\hat{\Sigma }$, which is given by Zeileis (2006) and is as follows:

$$\tilde{\Sigma }=\frac{1}{N}\hat{B}\hat{M}\hat{B},$$

where $\hat{B}$ is a block diagonal matrix, where the diagonal elements are the matrices $\left({\hat{B}}_{1},\text{…},{\hat{B}}_{J}\right)$ with ${\hat{B}}_{j}={\hat{I}}_{j}^{-1}N$ and the off diagonal elements are 0 and $\hat{M}=\frac{1}{N}{\hat{\Psi }}^{T}\hat{\Psi }$ with $\hat{\Psi }$ being an (N×JK) matrix of the score functions for the ith individual of the respective parameters. Normally, $\tilde{\Sigma }$ is the same as $\hat{\Sigma }$. However, for very large estimated standard errors, numerical limits may be reached within the statistical software R and implausible entries in the matrix may occur. This issue is addressed in Section 2.4.

In order to evaluate the hypotheses of interest simultaneously, we need a critical value against which we can compare our test statistics. We may choose a critical value $z_{1-\alpha ,M,\hat{R}}$ as the two‐sided quantile of a multivariate normal distribution with correlation $\hat{R}$. If $z_{1-\alpha ,M,\hat{R}}$ with index q=1,…,M, where M=JK are the elements of a multivariate normal random vector ${\mathrm{MZ}}_{M,0,\hat{R}}$ with expectation 0 and correlation matrix $\hat{R}$, then the critical value $z_{1-\alpha ,M,\hat{R}}$ is chosen such that $P(\max_{q=1,\text{…},M}\left(\right|z_{q}\left|\right)\leq z_{1-\alpha ,M,\hat{R}})=1-\alpha$. If an absolute test statistic is greater than the critical value, that is, $|t_{jk}|>z_{1-\alpha ,M,\hat{R}}$, we can reject the corresponding null hypothesis. This then allows us to calculate asymptotically adjusted p‐values for each of the hypotheses of interest by $p_{jk}^{MMM}=P(\max_{q=1,\text{…},M}\left(\right|z_{q}\left|)>\right|t_{jk}\left|\right)$. Further details on the calculation of the quantiles and probabilities of the multivariate normal distribution can be found in Genz and Bretz (2009) and Genz et al. (2021).

### 2.3 Bootstrap Approach

Another alternative to perform a multiplicity adjustment that will be examined is the vector‐based resampling approach proposed by Westfall and Young (1989). The p‐values obtained from the tests performed are adjusted to the case where all parameters $\beta_{jk}$ are equal to 0 for k>0. The single‐step adjusted p‐values ${\tilde{p}}_{jk}^{Bt}$ via bootstrapping can be defined as

$${\tilde{p}}_{jk}^{Bt}=P\left(\min\left(p^{*}\right)\leq p_{jk}\right),$$

where $p^{*}=\left(p_{11}^{*},\text{…},p_{JK}^{*}\right)$ is the p‐value vector from the respective resampled dataset. That means, ${\tilde{p}}_{jk}^{Bt}$ is the probability that the smallest p‐value of the resampled data is less than or equal to the observed unadjusted p‐value $p_{jk}$ in the original data. If we assume that the distributions of the endpoints are equal and possibly correlated, but the distributions and correlations are unknown we can approximate the adjusted p‐values ${\tilde{p}}_{jk}^{Bt}$ by resampling the individual row vectors. Row‐wise resampling automatically accounts for the correlation between endpoints without explicitly estimating it and implicitly accounts for the fact that binary data can be sparse and the lack of normality that is expected with small samples and rare binary events. The corresponding algorithm using bootstrap resampling is taken from Westfall and Young (1989) and is described below:

1. Compute the unadjusted p‐values $p_{jk}$ for the tests of interest.
2. Generate bootstrap data $y_{ji}^{*}$ by row‐wise resampling with replacement from the original data.
3. Compute a p‐value vector $p^{*}=\left(p_{11}^{*},\text{…},p_{JK}^{*}\right)$ from the bootstrap data $y_{ji}^{*}$ using the same tests as for the original data.
4. Note whether $\min(p^{*})$ is less than or equal to each of $p_{jk}$.
5. Repeat steps 2–4. B times, where B is the number of bootstraps.
6. Compute the proportions of the B samples for which $\min\left(p^{*}\right)<=p_{jk}$ for each of the original JKp‐values.

This procedure uses the minP statistic. An alternative would be the maxT statistic, which uses the maximum of the test statistic instead of the minimum of the p‐value. Westfall, Tobias, and Wolfinger (2011) recommended the minP method for binary data as the test statistics for such data can exhibit widely different means and variances, and therefore the use of the maxT statistic is inferior to the minP method.

It is also possible to obtain adjusted p‐values through permutation of the original data. The algorithm is the same, and the only difference is that the data are resampled without replacement instead of with replacement. Although the bootstrap method is said to be less accurate than the permutation method (Efron and Tibshirani 1993) and the type I error control is not guaranteed (Westfall 2011), it is still used for comparison because it is said to be applicable to a wider class of models, such as those with covariates (Bretz, Hothorn, and Westfall 2010).

### 2.4 Bayesian GLMs with Weakly Informative Priors

In the simulation study that follows, it is sometimes the case that only a very small number of events occur at one or more of the endpoints. This can lead to inflated and biased estimates of the coefficients and their standard errors when using a univariate GLM for that endpoint, known as sparse data bias or sparsity (Greenland, Schwartzbaum, and Finkle 2000; Peduzzi et al. 1997). Extreme coefficients and standard errors can also arise in the presence of separation in the data, including complete separation, where binary response variables can be perfectly distinguished based on predictors, and quasi‐complete separation, a less extreme form where predictors provide nearly perfect prediction for most predictor values (Albert and Anderson 1984).

To address the possible challenges posed by sparse data bias and separation, we propose the use of Bayesian generalized linear models (BGLMs). Gelman et al. (2008) state that these models provide more reasonable results in such situations. Thus, in addition to analyzing the data using GLMs, we applied BGLMs with weakly informative prior distributions. We used the same underlying model structures as for the GLMs (assumption of binomial distribution for the data, logit link, and either regression or group comparisons in the systematic part of the model) and fitted the BGLMs using the bayesglm function of the package arm (Gelman and Su 2021). We used the default settings of the function proposed by Gelman et al. (2008), which uses independent Cauchy distributions for all model parameters as the prior. All coefficients are centered at 0, and the scale parameter is 10 for the constant term (intercept) and 2.5 for all other coefficients. Prior to model fitting, binary inputs are centered at a mean of 0, and numeric inputs are scaled to a mean of 0 and a standard deviation of 0.5. To assess the extent to which BGLMs control the FWER and to compare their power with GLMs, we conduct a simulation study alongside GLMs, using BGLMs with identical parameter settings for all three methods. We have not investigated how the BGLMs behave when alternative values are used for the scale parameter.

In addition, a computational problem occurred in some cases where the application of GLMs resulted in extreme coefficients or standard errors due to sparsity at one or more endpoints: it was occasionally not possible to calculate the correlation matrix $\hat{R}$ using the multcomp package. This was due to negative entries in the diagonal of the variance–covariance matrix $\hat{\Sigma }$ of the stacked score contributions, which can occur when numerical limits are reached within the computational software. Consequently, p‐values could not be computed using the multivariate normal distribution with the MMM method, making it impractical to assess endpoints simultaneously using this approach in such situations.

Regarding the regression model, the evaluation of FWERW showed that this problem was most prevalent when the probability of occurrence was set to π=0.01, occurring in 1–50% of the simulations. However, a high number of problems were observed in only a few parameter settings with small sample sizes and a large number of endpoints. At a probability of occurrence of π=0.0625, the issue was largely mitigated and only a few parameter settings caused problems in up to 3% of the simulations. In simulations assessing FWERS and power for the regression model, the problem only surfaced when N=100 and only in up to 0.2% of the simulations.

For group comparison models, the problem occurred more frequently, especially when a dose group had no or very few events. When the probability of occurrence at an endpoint was only π=0.002, the problem occurred in 28% to even 94% of simulations. However, when the probability of occurrence was at least π=0.01, the problem was greatly reduced, occurring in only a few settings in up to 28% of simulations, but for most settings in less than 5% of simulations.

While it could be argued that endpoints with sparse data are not necessarily relevant for real data analysis due to the lack of events, the determination of a threshold for endpoint removal remains unclear. Furthermore, the use of a model that provides more plausible results for named situations may be advantageous, especially for less supervised analyses. Also, as mentioned above, separation can lead to the same problems, and it would not make sense here to simply exclude endpoints where such a problem occurs. As a further alternative to deal with separation and sparse data, the brglm function (Kosmidis 2021) can be considered. It is based on the bias reduction method developed by Firth (1993), and the resulting estimates and their corresponding standard errors should always be finite (Kosmidis and Firth 2020).

### 2.5 Simulation Study

**FIGURE A1:**
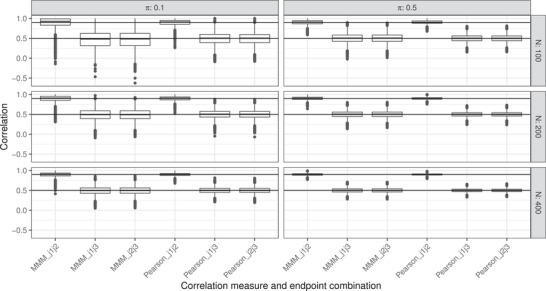
Specified and estimated correlation between 3 endpoints. The x‐axis shows the corresponding combinations of endpoints and the associated method used to determine the correlation. The columns indicate the probabilities of occurrence in all endpoints and the rows indicate the sample sizes.

A simulation study was conducted to investigate and compare the FWER and power of the proposed p‐value adjustments using regression and group comparison models with various parameter settings. We considered the any‐pair power, which is defined as the probability of detecting at least one correct rejection of a null hypothesis (Ramsey 1978). The global two‐tailed significance level was set to α=0.05. All simulations were carried out in R version 4.1.1 (R Core Team 2021). The MultiRNG package (Demirtas, Allozi, and Gao 2021) was used to generate correlated binary data using the method described in Park, Park, and Shin (1996). A simulation of 5000 runs (results shown in Figure A1 in the Appendix) shows that this method achieves on average the prespecified correlation between the endpoints. The multcomp package (Hothorn, Bretz, and Westfall 2008) and the mmm and glht functions were used to perform the p‐value adjustment using the MMM approach. For each combination of settings and methods, 10,000 simulations were run to estimate FWER and power. For the bootstrap approach, B=1000 bootstrap samples were drawn per simulation. In general, the correlation between the binary endpoints was set to 0, 0.5, and 0.9. However, the data from the endpoints where all parameters were under the null hypothesis and the endpoints where some parameters were under the alternative hypothesis were sampled independently with the correlations. This means that the correlation between these two groups of endpoints was not specified and known.

For the assessment of FWERW in the regression model, scenarios with J∈{3,5,10} endpoints were examined and we used N∈{100,200,400} observations divided into four arbitrarily chosen dose levels x∈{0,2.5,5,10} with balanced sample sizes. For $\beta_{j0}=\log\left(\pi /1-\pi \right)$, which describes the intercept in the regression model and thus also the general probability of occurrence for each endpoint on the logit scale for the case without dose effects, the probability of occurrence was set to π∈{0.01,0.0625,0.125,0.25,0.375,0.5}. The slope was set to $\beta_{j1}=0$ for all parameter settings.

To evaluate the power and the FWERS in the same model, a total of J=10 endpoints were always considered. Either 2 or 8 endpoints were under the alternative hypothesis, and the remaining endpoints were under the null hypothesis. The same dose levels and sample size N were used as in the previous simulation, and the intercept $\beta_{j0}=\log\left(\pi /1-\pi \right)$, corresponding to π=0.1 at dose level x=0 was chosen for all settings. Nine different slope parameters $\beta_{j1}\in \left\{0.001,0.025,0.05,0.075,0.1,0.125,0.15,0.175,0.2\right\}$ were chosen for the endpoints under the alternative, while for the endpoints under the null, the slope parameter was always set to $\beta_{j1}=0$.

For the simulation with the group comparison model, a total of 43 different settings were selected. These were chosen to reflect real situations as closely as possible. A total of 10 endpoints were always considered, and the sample size was set to N∈{100,200}. Three settings were chosen in which the parameters of all endpoints were under the null hypothesis, but three or five endpoints had different intercepts $\beta_{j0}$. In the remaining 40 settings, all parameters of either two or eight endpoints were set under the null hypothesis, and the parameters of the remaining endpoints were set in various different configurations. An overview of the settings is given in Tables A1 and A2 in the Appendix.

**TABLE 1:** Adjusted p‐values for each tumor endpoint using the three different methods and regression models with the two model classes for the NTP bioassay on methyleugenol. The B in front of the method name indicates that a BGLM was used instead of a GLM.

Endpoint	Bonferroni	B_Bonferroni	MMM	B_MMM	Bootstrap	B_Bootstrap
t24	0.0227	0.0269	0.0221	0.0260	0.0120	0.0151
t26	1.0000	1.0000	0.9987	0.9989	0.9995	0.9995
t27	0.0032	0.0036	0.0031	0.0035	0.0015	0.0014
t28	1.0000	1.0000	1.0000	1.0000	1.0000	1.0000
t29	0.0000	0.0000	0.0000	0.0000	0.0000	0.0000
t36	0.6558	0.7304	0.4730	0.5106	0.4486	0.4940
t41	0.2386	0.2684	0.2066	0.2290	0.1723	0.1859
t42	0.0000	0.0000	0.0000	0.0000	0.0000	0.0000
t59	0.0066	0.0071	0.0065	0.0070	0.0028	0.0031
t71	0.4336	0.2214	0.3437	0.1932	0.3106	0.1520

When using GLMs and the MMM approach, the null hypothesis was counted as not rejected for those simulation runs, where the calculation of the correlation matrix was not possible due to the problem described in Section 2.4. This does slightly influence the simulation results, as all hypotheses of all endpoints count as rejected only because of one or a few poorly behaved endpoints. Therefore, the GLM combined with the MMM method looks more conservative in settings where this problem frequently occurs.

## 3 Results

### 3.1 Regression Model

**FIGURE 1:**
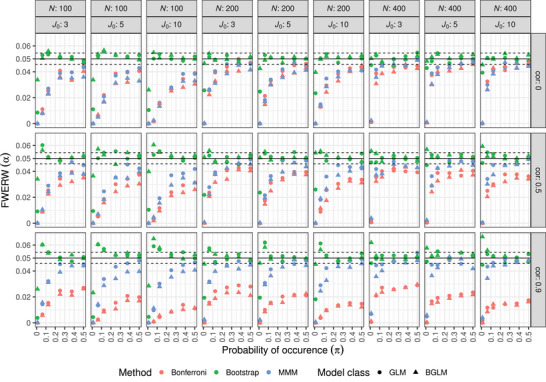
Simulated FWERW for the three methods considering regression models where all hypotheses are under the null. The rows depict the correlation between the endpoints and the columns depict the sample size (N) and the number of endpoints ($J_{0}$) examined. The shape represents the two model classes. The a priori chosen α of 0.05 is represented by the horizontal line and the dashed lines represent the standard error of the simulation.

First, the results of the regression model are presented. Figure 1 shows the estimated FWERW for the three p‐value adjustment procedures. It can be seen that the Bonferroni and MMM methods control the FWERW for all parameter settings. For the bootstrap method, the FWERW is controlled for almost all settings, and only when the probability of occurrence and the sample size becomes smaller and the correlation between the endpoints becomes larger, the nominal level of 0.05 is sometimes slightly exceeded. The Bonferroni procedure is below 0.05 for almost all settings and is almost always the most conservative procedure. In the absence of correlation, the Bonferroni and the MMM methods have very similar FWERWs. However, as the correlation between the endpoints increases, the Bonferroni method becomes strongly conservative, while the MMM approach gets closer to the nominal level. In general, it can be seen that the MMM method approaches the nominal level as the sample size increases. Comparing the application of BGLMs with the application of GLMs, it is noticeable that the BGLMs are almost consistently more conservative than the GLMs in the case of the Bonferroni and MMM methods, and only for a few settings is the FWERW slightly more liberal. Using the bootstrap approach, the two model classes appear to lead to a similar FWERW, except for cases with rare events where the BGLMs are less conservative.

**FIGURE 2:**
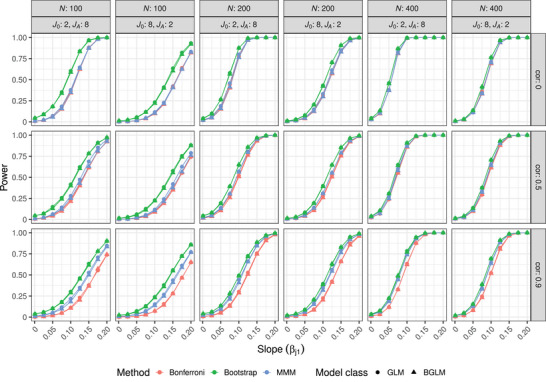
Simulated power for the three methods considering regression models with either two or eight hypotheses under the alternative. The rows depict the correlation (cor) between the endpoints and the columns depict the sample size (*N*) and the combination of endpoints under the null and alternative ($J_{0}$, $J_{A}$). The shape represents the two model classes.

The results of the power simulations for different parameter settings are shown in Figure 2. It is noticeable that the bootstrap approach shows a power gain for all parameter settings compared to the other two methods. For a correlation of 0 and a sample size of 100, the advantage reaches up to 25% in terms of power gain, and for a correlation of 0.5 the gain is still up to 13% compared to the MMM method. As the sample size increases, the gain of the bootstrap method decreases compared to the MMM method. In general, the performance gain of both methods compared to the Bonferroni method is evident when the correlation between the endpoints is high. Regarding the model classes, the BGLMs have very similar chances of detecting differences as the GLMs and show slightly lower power on average.

**FIGURE 3:**
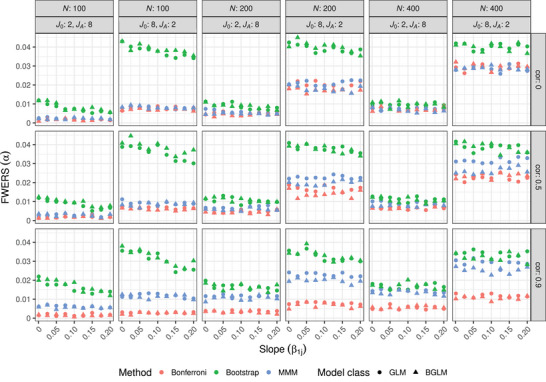
Simulated FWERS for the methods considering regression models where all either two or eight hypotheses are under the alternative. The rows depict the correlation between the endpoints (cor) and the columns depict the sample size (*N*) and the combination of endpoints under the null and alternative ($J_{0},J_{A}$). The shape represents the two model classes.

The power simulation was also used to see if the FWER is controlled in a strong sense. Figure 3 shows that all three methods and both model classifications control the FWERS. The bootstrap approach is always less conservative than the other two but behaves more similarly when the number of endpoints under the alternative hypothesis and the sample size are large. While the MMM and Bonferroni methods behave quite similarly when there is no correlation between the endpoints, the Bonferroni method becomes more conservative as the correlation increases. Again, looking at the MMM and Bonferroni methods, we can see that BGLMs are more conservative than GLMs. However, when using the bootstrap method, no differences or patterns can be noted.

### 3.2 Group Comparison Model

**FIGURE 4:**
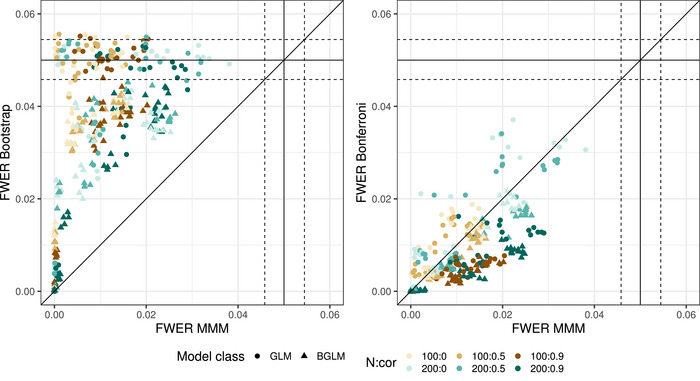
Simulated FWERs for the MMM method (*x*‐axis) plotted against the bootstrap and Bonferroni method. Colors indicate the combination of sample size and correlation between the endpoints; symbols distinguish the two model classes. Dashed horizontal and vertical lines show the range in which 95% of all simulation results (10,000 simulations) would fall if a method had exactly 5% true FWER.

For the results of the simulation with the group comparison model, the MMM method was compared with the other two methods in terms of FWER and the power. Figure 4 shows the FWER comparison for all simulated parameter settings. It can be seen that the MMM method is always more conservative compared to the bootstrap, and the bootstrap has FWERs up to 0.05 for many settings and rarely behaves slightly liberal. It is also apparent that the MMM method yields slightly less conservative FWERs as the correlation between the endpoints increases. Looking at the comparison between MMM and Bonferroni, the difference is not quite as strong. Both methods have a rather conservative FWER and as the correlation between the endpoints increases, the FWER of the MMM method becomes slightly higher than that of the Bonferroni method. For some settings with the GLMs, the Bonferroni seems to be less conservative than the MMM method. An investigation showed that these are settings where the probability of occurrence in some groups is very low, and in many simulation runs it was not possible to estimate the correlation matrix between the parameters with the MMM method. Since in such cases the null hypothesis was counted as not rejected, the MMM method appears to be more conservative.

**FIGURE 5:**
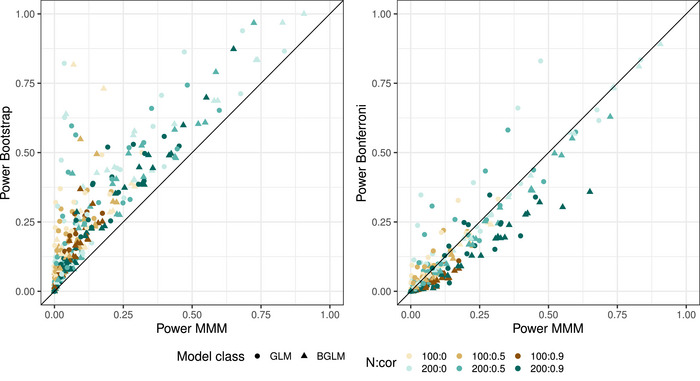
Simulated power for the MMM method (*x*‐axis) plotted against the bootstrap and Bonferroni method. Colors indicate the combination of sample size and correlation between the endpoints; symbols distinguish the two model classes.

Figure 5 shows the power of the MMM method compared to the power of the other two methods. It is clear that the bootstrap has a higher power than the MMM method in all the settings examined. The gain can be up to 70% in some cases. In many settings, a higher sample size leads to a slightly smaller gap between the bootstrap and the MMM method. Comparing MMM and Bonferroni, the performance is very similar when the correlation is low and the MMM method shows its advantage when the correlation between the endpoints is higher. In some cases, when considering the GLM, the Bonferroni seems to outperform the MMM method. This is again due to the fact that sometimes the correlation matrix cannot be estimated when the probability of occurrence in some groups is very low, and therefore the null hypothesis cannot be rejected. Further examination of the results by the authors also showed that BGLMs outperformed GLMs in some settings. There was a tendency for BGLMs to show an advantage when a larger dose effect was simulated. However, no clear pattern emerged.

The raw data for all simulations and more detailed graphs for the group comparison model, where the corresponding settings can be seen, are available on GitHub at https://github.com/sbudig/glm_mult_bindat.

## 4 Example

**FIGURE 6:**
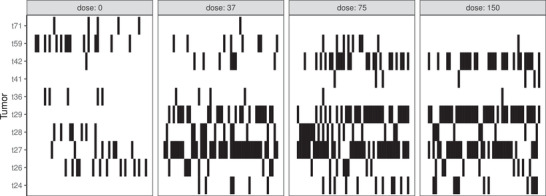
Presence (black) and absence (white) of 10 tumor classes in 200 mice after 2 years of receiving methyleugenol at four different concentrations.

In a bioassay conducted by the National Toxicology Program (NTP 2000), the toxicity of methyleugenol was investigated using female mice. Methyleugenol is used as an additive in many food and cosmetic products. Groups of 50 mice were given methyleugenol in 0.5% methylcellulose at doses of 0, 37, 75, or 150 mg/kg for 105 weeks, and tumor incidence was assessed at a total of 89 tumor sites. Hothorn (2016) had already performed a control‐versus‐treatment analysis on the underlying dataset using a permutation approach and the MMM method. However, the analysis focused only on liver tumor sites. We restricted the analysis to the 10 tumor classifications where a tumor occurred more than five times in total across all mice. The presence or absence of the 10 selected tumor classifications at the four dose levels is shown graphically in black and white in Figure 6. It can be seen that tumor classifications t26, t27, t28, and t29 are negatively correlated with each other, that is, only one of the two classifications can occur. This is due to the fact that they are subclasses of the same tumor.

For each of the 10 tumors, a binomial regression model was fitted to determine whether increasing doses of methyleugenol promoted the development of certain tumors. To comply with the FWER, the p‐values for the respective dose slopes were then adjusted using the three methods described. For the bootstrap method, the dataset was resampled 10,000 times. Models fitted with BGLMs were also considered.

The adjusted p‐values are shown in Table 1. When the p‐values are evaluated with a 5% probability of error, there are no differences in significance between the three procedures and the two model classes. However, it can be seen that the Bonferroni correction consistently gives the most conservative p‐values. The bootstrap approach gives the smallest p‐values, and the MMM approach is in between the two. Except for tumor t71, the BGLMs provide more conservative p‐values than the regular GLMs.

Example R code for the analysis of the described NTP bioassay using regression models and the three methods, and the resulting effect estimates and standard errors are available from the aforementioned GitHub link. We also provide the code, estimators, and adjusted p‐values from the analysis of the NTP bioassay using a group comparison model, which can be used to compare the dose levels with the control. It should be noted that extreme coefficients and standard errors occur for tumors t24, t29, t41, and t71 when using a GLM with group comparison models, as no tumors occur at some dose levels. In this case, the MMM method can, therefore, no longer be used to adjust the *p*‐values of all tumors considered together, as no correlation matrix can be calculated. The use of BGLMs provides a remedy for this and also leads to considerably lower p‐values with the Bonferroni and bootstrap in some cases compared to the GLMs.

## 5 Discussion

To date, only limited attention has been paid to the problem of multiple testing when analyzing multivariate binary data. We have applied the MMM approach of Pipper, Ritz, and Bisgaard (2012) to allow simultaneous inference for multiple binary endpoints. It is a flexible approach that takes into account the correlation between the parameters without requiring the explicit specification of a correlation structure. We compared the method in terms of FWER and power with two other methods in a simulation study, as it was not clear how the asymptotic method performed with small sample sizes and what advantage it might provide when endpoints are correlated. The simple Bonferroni correction, which is known to be very conservative in terms of the FWER, was used for comparison. In addition, the bootstrap approach with minP adjustment of Westfall and Young (1989) was considered because, although this procedure has been known for a long time, it was not clear how it would perform with binary data.

The specific settings of the simulations are motivated by long‐term carcinogenicity studies. However, datasets of multivariate, correlated binary endpoints with limited sample sizes are also likely to be encountered in other toxicological applications of preclinical studies or chemical risk assessments. Another important application with similar data characteristics is the assessment of adverse events in early‐phase clinical trials.

Our simulation study shows that for both the regression and group comparison models, all three methods control the FWER in both the weak and strong sense for almost all parameter settings typical of toxicology or carcinogenicity studies.

The Bonferroni correction is consistently the most conservative method and lacks power, but in the absence of correlation between endpoints the MMM method performs equally. In contrast to the large sample application in So and Sham (2011), we found that the bootstrap method can improve power for binary data with small sample sizes. It consistently outperforms the other two methods, especially when considering group comparison models and it almost always controls the FWER in both the weak and strong sense. We think that there are at least two reasons for this performance. First, the bootstrap does not rely on the normal approximation when calculating the adjusted p‐values. By using a simulated distribution of p‐values, the lack of normality of the test statistics is at least partially compensated for. This is most important when the expected number of events is small (due to small proportions and/or small numbers of cases). Second, when endpoints have only very few events, they may not lead to any rejection of the null hypothesis when resampling, and therefore no type I error can occur. This means that these endpoints never contribute to extreme test statistics and therefore cannot contribute to a minimum p‐value. They are, therefore, effectively excluded from the p‐value adjustment without being formally excluded from the procedure. The MMM and the Bonferroni methods, on the other hand, always assume a normal distribution of the *p*‐values/test statistics and always consider the full number of endpoints that were formally included in the analysis. However, as the sample size and correlation increase, the difference in performance between the MMM and the bootstrap method decreases slightly in terms of power.

It can be concluded that both the bootstrap and the MMM methods are preferable to the Bonferroni correction in all parameter settings considered. And even if the bootstrap always seems to be superior, a custom code has to be written for the analysis (see Supporting Information) and the MMM method has a number of advantages over previous software implementations and the bootstrap approach: First, it is easy to extend the multiple comparisons to pairwise tests between the dose groups, rather than just testing a treatment effect versus the control. Schaarschmidt, Ritz, and Hothorn (2021) provide details in a more general context, including extensions to more general multiple contrast tests for testing dose–response relationships. However, with multiple comparisons between dose groups, computational problems associated with rare binary events (Hauck–Donner effect) become more prevalent than when testing a single regression slope for each endpoint. For these extensions, approaches that provide stable estimates in small sample and rare event settings, such as weakly informative Bayesian GLMs (Gelman et al. 2008) or bias‐reduced GLMs (Kosmidis and Firth 2020), become more important. Second, in addition to the adjusted p‐values, the computation of asymptotic simultaneous confidence intervals is straightforward with the MMM approach. These can be of interest when assessing effect sizes, rather than just assessing the significance of effects for each endpoint. They also allow the assessment of noninferiority or equivalence for multiple binary endpoints compared to an untreated control, as long as the testing problem is defined as a union‐intersection test (termed local noninferiority by Hasler and Hothorn (2013)). Simultaneous confidence intervals for multivariate binary data are not directly available for the resampling approach of Westfall and Young (1989) or the exact or permutation tests implemented by Hothorn et al. (2006).

Finally, the MMM approach easily allows modeling dose–response effects in the presence of further covariates or additional categorical predictor variables (Pipper, Ritz, and Bisgaard 2012; Schaarschmidt, Ritz, and Hothorn 2021). These may be subject specific, numerical covariates, but may also model differences between subgroups or strata of the population, such as gender, age groups, or different levels of exposure to other risk factors. For bootstrap approaches or exact permutation tests for multiple binary endpoints (Ristl et al. 2019; Westfall and Young 1989), such extensions are currently not described and it is unclear what the resampling scheme should look like.

In this paper, we have focused on single‐step p‐value adjustments. One way to increase the average power, that is, the ratio of the number of correct rejections to the total number of hypotheses for which the alternative applies would be to use stepwise procedures. He and Heyse (2019) describe three step‐down procedures for discrete data. One of these uses the permutation distribution and thus takes into account possible correlations between endpoints. This results in higher average power than the bootstrap method presented here. No step‐up procedure for discrete data that takes into account possible correlations and thus offers an advantage has been found. In general, simultaneous confidence intervals are more difficult to obtain and interpret when derived in a manner consistent with stepwise multiplicity adjustment approaches (Strassburger and Bretz 2008).

If the occurrence of an event is very rare, the coefficients and standard errors estimated with GLMs can become extremely large. As a result, sometimes no correlation matrix between the model parameters can be calculated with the multcomp package, and thus no adjusted p‐values can be generated with the MMM method. Here we have shown that the use of BGLMs can be an alternative in this case, since the parameter space is limited by the a priori assumptions, and thus the estimation of the standard errors is more stable when the occurrence of an event is very rare (Gelman et al. 2008). In general, the simulations showed that the price of a more stable estimate was somewhat more conservative behavior, especially in regression. However, the bootstrap showed little difference between the model classes and there were situations, for example, when the dose effect in the group comparisons was large, where the use of BGLMs could represent a performance gain. However, no clear pattern could be identified.

Another approach to mitigate the problem of extreme coefficients or standard errors would have been to set a threshold for the number of events required for each endpoint to be analyzable and thereby remove endpoints that fall below this threshold. However, determining the exact threshold is not straightforward. Vittinghoff and McCulloch (2006) conducted an extensive simulation study to investigate the minimum number of events per predictor variable required for meaningful analysis in logistic and Cox regression. Their results showed that with two to four events per predictor variable, there is a notable increase in bias and confidence interval coverage of less than 93%. Such results could be used to establish a threshold that should minimize the sparsity problem. An alternative approach that avoids omitting available data is to combine several rare event endpoints into a composite endpoint that is retained in the analysis. Then, if these endpoints are similarly affected by an increase in dose, such an effect would be more likely to be detected.

The methods outlined in this paper aim to control the probability of at least one false rejection when the objective is to show that at least one of several binary endpoints shows a dose–response relationship. However, in many applications of toxicological or clinical research, the primary aim is to prove the opposite: namely, that there is no dose–response relationship, that is, that there is no (relevant) change in the proportion of tumors, acute toxicity symptoms, adverse events, etc. In such situations, it would be counterintuitive to test for significant dose–response relationships and additionally use methods to adjust for multiple comparisons. Applying a multiplicity correction in such cases would increase the chance of failing to detect an existing dose–response relationship. We, therefore, advise against using any of the methods described in this paper in such situations. Rather, if the aim is to show no relevant effect, methods for assessing equivalence or noninferiority would be appropriate.

## Conflicts of Interest

The authors declare no conflicts of interest.

## Open Research Badges

This article has earned an Open Data badge for making publicly available the digitally‐shareable data necessary to reproduce the reported results. The data is available in the Supporting Information section.

This article has earned an open data badge “**Reproducible Research**” for making publicly available the code necessary to reproduce the reported results. The results reported in this article were reproduced partially due to missing seeds of the RNG and computational complexity.

## Supporting information

Data S1

## Data Availability

The data that supports the findings of this study are available in the supplementary material of this article.

## Appendix

### A1 Generation of Correlated Binary Endpoints

To generate correlated binary data, the approach of Park, Park, and Shin (1996) was chosen in this paper. The MultiRNG package (Demirtas, Allozi, and Gao 2021) was used for this purpose. In a small simulation of 5000 runs, it was examined whether the specified correlations between the endpoints are achieved. The simulation was carried out with three endpoints. The correlation between endpoints $j_{1}$ and $j_{2}$ was set to 0.9. Between endpoints $j_{1}$ and $j_{3}$ and $j_{2}$ and $j_{3}$, the correlation was set to 0.5. For $\beta_{j0}=\log\left(\pi /1-\pi \right)$, which describes the intercept in the regression model, the probability of occurrence was set to π∈{0.1,0.5}. The slope was set to $\beta_{j1}=0$ for all parameter settings, meaning that there were no different probabilities of occurrence between the dose groups. The correlation between the endpoints was then calculated once according to Pearson and once estimated using the MMM method. Figure A1 shows the result of this simulation. It can be seen that, on average, the correlation between the endpoints is achieved in the settings examined. In some cases, a slight leftward skew can be seen, which becomes more pronounced when the correlation between the endpoints is 0.9. The variation in the estimated correlation using Pearson appears to be smaller than the estimated correlation using the MMM method.

### A2 Group Comparison Model Settings

For the group comparison model simulation, 43 different parameter settings were selected. Table A1 lists 40 of these settings. Here, $J_{0}$ indicates the number of endpoints where all parameters were in line with the null hypothesis, and the number of endpoints where some parameters were under the alternative hypothesis is given by $J_{A}$. The probability of occurrence for the $J_{0}$ endpoints is indicated by $\pi_{0}$ with $\beta_{jk}=\log\left(\pi_{0}/1-\pi_{0}\right)$ for all parameters. For the $J_{A}$ endpoints, the probability of occurrence for the intercept or the control group is indicated by $\pi_{A}$ with $\beta_{j0}=\log\left(\pi_{A}/1-\pi_{A}\right)$. First, different slopes were used to simulate an increase in the dose groups. The corresponding value in the column *Slope* indicates how much the probability on the logit scale increases when the dose level is increased by 1 and the arbitrary dose levels {0,2.5,5,10} were chosen. Second, only an increase in the last dose group was simulated with respect to the remaining dose groups. The value in the column $\pi_{k0-k3}$ indicates the difference between the probabilities of occurrence of $\beta_{j3}$ and the other three parameters $\beta_{jk}$ with k=0,1,2. For all settings, the sample size was set to N∈{100,200}.

In addition, three further settings were simulated in which all *s* of all endpoints were in line with the null hypothesis, but there were different probabilities of occurrence between the endpoints. Table A2 shows the three settings with the corresponding probabilities of occurrence for the individual endpoints. The columns reflect the different endpoints.

**TABLE A1:** Different parameter configurations where parameters of endpoints are either under the alternative or under the null for the simulation using the group comparison model.

$J_{0}$	$\pi_{0}$	$J_{A}$	$\pi_{A}$	Slope	$\pi_{k0-k4}$
2	0.02	8	0.4	0.1	—
2	0.1	8	0.4	0.1	—
2	0.4	8	0.02	0.1	—
2	0.4	8	0.1	0.1	—
2	0.02	8	0.4	0.2	—
2	0.1	8	0.4	0.2	—
2	0.4	8	0.02	0.2	—
2	0.4	8	0.1	0.2	—
2	0.02	8	0.4	0.3	—
2	0.1	8	0.4	0.3	—
2	0.4	8	0.02	0.3	—
2	0.4	8	0.1	0.3	—
2	0.02	8	0.4	—	0.1
2	0.02	8	0.4	—	0.2
2	0.1	8	0.4	—	0.1
2	0.1	8	0.4	—	0.2
2	0.4	8	0.02	—	0.1
2	0.4	8	0.1	—	0.1
2	0.4	8	0.02	—	0.2
2	0.4	8	0.1	—	0.2
8	0.02	2	0.4	0.1	—
8	0.1	2	0.4	0.1	—
8	0.4	2	0.02	0.1	—
8	0.4	2	0.1	0.1	—
8	0.02	2	0.4	0.2	—
8	0.1	2	0.4	0.2	—
8	0.4	2	0.02	0.2	—
8	0.4	2	0.1	0.2	—
8	0.02	2	0.4	0.3	—
8	0.1	2	0.4	0.3	—
8	0.4	2	0.02	0.3	—
8	0.4	2	0.1	0.3	—
8	0.02	2	0.4	—	0.1
8	0.02	2	0.4	—	0.2
8	0.1	2	0.4	—	0.1
8	0.1	2	0.4	—	0.2
8	0.4	2	0.02	—	0.1
8	0.4	2	0.1	—	0.1
8	0.4	2	0.02	—	0.2
8	0.4	2	0.1	—	0.2

**TABLE A2:** Different parameter configurations where all parameters of the endpoints are under the null for the simulation with the group comparison model. The columns show the individual endpoints with the respective probabilities of occurrence for each setting.

1	2	3	4	5	6	7	8	9	10
0.001	0.001	0.125	0.125	0.25	0.25	0.375	0.375	0.5	0.5
0.001	0.001	0.001	0.001	0.325	0.325	0.325	0.5	0.5	0.5
0.1	0.1	0.1	0.1	0.3	0.3	0.3	0.5	0.5	0.5
